# Clinical Experience with URO17^®^ in the Diagnosis and Surveillance of Bladder Cancer

**DOI:** 10.3390/jcm14228108

**Published:** 2025-11-16

**Authors:** Shahram Shawn Gholami, Mehran Movassaghi, Sasha Homayoun, Nikhil Vasdev

**Affiliations:** 1 Urology Association of Silicon Valley, San Jose, CA 95124, USA; 2 Department of Urology, Providence Saint John’s Health Center, Santa Monica, CA 90404, USA; 3 Department of Mathematics, Southern New Hampshire University, Hooksett, NH 03106, USA; 4 Department of Urology, East and North Hertfordshire NHS Trust, Lister Hospital, Stevenage SG1 4AB, UK

**Keywords:** bladder cancer, cystoscopy, hematuria, keratin 17 (K17), non-muscle-invasive bladder cancer (NMIBC), noninvasive diagnostics, urothelial carcinoma, URO17^®^, urine cytology, urinary biomarker

## Abstract

**Objective:** To describe the clinical use of URO17^®^, a noninvasive, urine-based immunocytochemistry assay targeting Keratin 17 (K17), as an adjunct to conventional diagnostic methods for urothelial carcinoma. **Materials and Methods:** These illustrative cases summarize the real-world use of URO17^®^ in diagnostic workflows for patients presenting with hematuria and those undergoing surveillance for non-muscle invasive bladder cancer (NMIBC). Urine samples were processed via standard immunocytochemistry and interpreted alongside cystoscopic, cytological, and radiographic findings. **Discussion:** URO17^®^ was used as a complementary diagnostic tool to help guide clinical management. Negative results supported deferral of invasive procedures in selected patients, while positive findings prompted further evaluation when standard tests were inconclusive. **Conclusions:** In the seven illustrative cases presented, URO17^®^ aided clinical decision-making as part of routine diagnostic and surveillance workflows. The test’s integration with existing cytology processes supports its potential role as a noninvasive adjunct for evaluating patients with suspected or recurrent urothelial carcinoma.

## 1. Introduction

Bladder cancer is the 10th leading cause of cancer death in the United States, representing a persistent challenge for both patients and clinicians. In 2025, an estimated 84,870 new cases and approximately 17,420 deaths are expected nationwide [1]. A hallmark of bladder cancer, particularly non-muscle invasive disease, is its high recurrence rate. Even after successful tumor removal, recurrence can occur in up to ~75% of cases [2]. This necessitates ongoing surveillance and, in many cases, additional intravesical therapy, placing a significant burden on patients and the healthcare system.

Traditional diagnostic tools such as cystoscopy and urine cytology have long served as the cornerstone of bladder cancer evaluation, but both present notable limitations. Urine cytology, while highly specific, demonstrates variable but generally poor sensitivity-reported between 45% and 55% overall and significantly lower for low-grade tumors [3]. It frequently yields ‘atypical’ or ‘suspicious’ results, creating diagnostic uncertainty and delaying clear treatment planning [3]. Cystoscopy, though more sensitive (approximately 71%), remains invasive, uncomfortable, and costly, with false-negative rates as high as 25–35% [4]. It also lacks the ability to assess upper urinary tract lesions and may involve prolonged wait times before evaluation.

To address these diagnostic gaps, URO17^®^ has emerged as a noninvasive, urine-based immunocytochemistry assay that detects Keratin 17 (K17), a biomarker overexpressed in urothelial carcinoma cells. The test provides binary results based on an established cut-off, positive or negative, allowing for straightforward clinical interpretation.

This report presents a set of representative real-world cases that illustrate how, in our experience, URO17^®^ has facilitated earlier detection, guided treatment decisions, and improved diagnostic confidence in both initial hematuria evaluations and surveillance of non-muscle-invasive bladder cancer.

## 2. Materials and Methods

### 2.1. URO17^®^ Integration in Clinical Pathways

**Figure 1:**
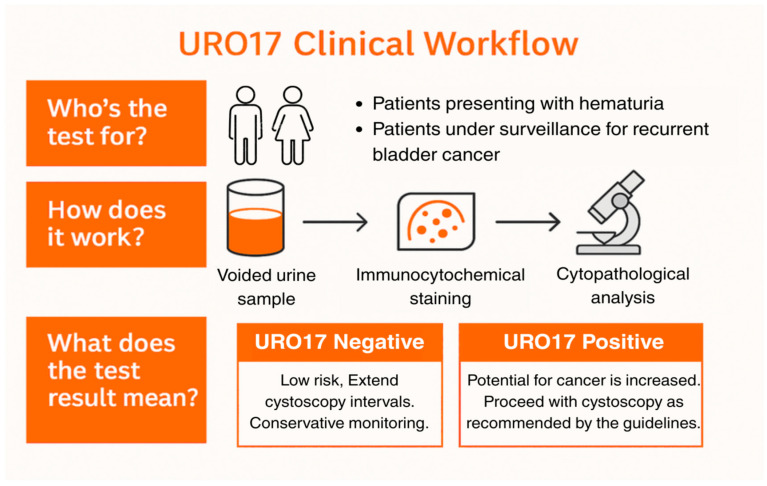
URO17^®^ Clinical Workflow for Diagnostic and Surveillance Pathways. Schematic representation of the URO17^®^ urine immunocytochemistry (ICC) workflow. Voided urine samples from patients presenting with hematuria or under surveillance for recurrent non–muscle-invasive bladder cancer (NMIBC) are processed using ICC for Keratin 17 (K17). Results are binary—URO17^®^ Negative (low likelihood of urothelial carcinoma; patients may continue with conservative monitoring or extended cystoscopy intervals) or URO17^®^ Positive (increased likelihood of malignancy; cystoscopy is recommended according to standard guidelines). This figure illustrates the conceptual workflow used in these cases and is not data derived.

**Figure A1:**
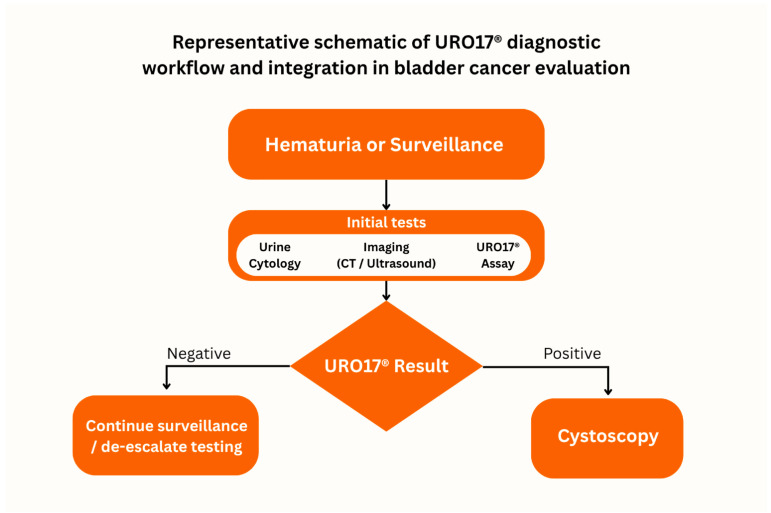
Representative schematic of URO17^®^ diagnostic workflow and integration in bladder cancer evaluation. Patients presenting with hematuria or under surveillance for recurrent bladder cancer undergo standard evaluations, including urine cytology and imaging. URO17^®^ results then inform next steps: a positive result prompts cystoscopy, whereas a negative result supports continued surveillance or de-escalation of invasive testing. This figure is an original creation by the authors and is illustrative rather than data derived.

URO17^®^ testing was incorporated into diagnostic and surveillance pathways for patients presenting with hematuria or undergoing follow-up for non-muscle-invasive bladder cancer (NMIBC). The general diagnostic and surveillance workflow is illustrated in Figure 1, and a representative schematic provided in Appendix A (Figure A1). This report describes a curated set of seven real-world cases that spanned the years 2019–2024 across multiple urology practices, in which URO17^®^ was used to support clinical decision-making as part of routine practice. Inclusion criteria consisted of patients with available URO17^®^ test results accompanied by corresponding cytology, cystoscopy, or imaging findings that allowed complete diagnostic correlation. No formal exclusion criteria were applied other than incomplete diagnostic documentation. Cases were selected retrospectively for their illustrative value in demonstrating varied clinical applications rather than enrolled consecutively and thus represent a descriptive, experience-based series rather than a systematic study.

### 2.2. Urine Cytology Method

Specimens arrived preserved in a ratio of 2:1, Voided Urine to PreservCyt (Hologic) from the clinics to the testing laboratory. 50 mL voided urine samples were centrifuged at 2500 rpm for 5 min, then the gently suspended pellets were filtered through polycarbonate membrane filters (ThinPrep™ 5000 processor, HOLOGIC). Cell monolayers were obtained by gently imprinting the filters onto glass slides. The samples were fixed by immediate immersion in Delaunay fixative (96% ethanol 1:1 + 0.5 mL/L trichloroacetic acid) and then stained with Papanicolaou. Based on the Paris system diagnostic criteria, the samples were diagnosed as High-Grade Urothelial Carcinoma (HGUC), Suspicious of High-Grade Urothelial Carcinoma (SHGUC), Atypical Urothelial Cells (AUC), and Negative for High Grade Urothelial Carcinoma (NHGUC) [5].

### 2.3. URO17^®^ Immunocytochemistry

Samples were centrifuged at 1000× *g* for 10 min; each pellet was resuspended in 20 mL of PreservCyt (Hologic) and then transferred to charged-glass slides using a T-5000 (Hologic) cell processor. The slides were stained using a Link 48 Autostainer (Agilent Technologies, Santa Clara, CA, USA). Endogenous peroxidase activity was blocked using the EnVision FLEX wash peroxidase-blocking reagent (Agilent Technologies). Slides were incubated with anti-Acu-URO17^®^ antibody (KDx1 mAb; 1:32 dilution), processed with the direct polymer-based immunoperoxidase method using EnVision FLEX HRP, developed in EnVision FLEX DAB+ chromogen, and counterstained with hematoxylin. Slides were dehydrated with graded ethanol and protected with a cover slip. A pathologist screened the slides, quantifying the total number of urothelial cells expressing URO17^®^ per slide [5]. URO17^®^ slides were independently scored and the number of cells expressing URO17 were reported using the following criteria:

- Negative: fewer than 5 URO17^®^-positive urothelial cells.
- Positive: 5 or more URO17^®^-positive urothelial cells.

Turnaround time for URO17^®^ results in this series typically ranged from 24 to 48 h. URO17^®^ findings were interpreted alongside cytology, cystoscopy, and imaging results to inform subsequent management decisions. The test was used to clarify ambiguous cytology findings, triage hematuria workups, and guide escalation or de-escalation of care in appropriate cases. Each case reflects the clinical judgment of the treating physician and demonstrates how URO17^®^ results contributed to individualized management decisions.

## 3. Discussion

### 3.1. Overview of Cases and Testing Volume

Across seven real-world patient cases, a total of 13 URO17^®^ immunocytochemistry assays were performed (*n* = 13) across both diagnostic and surveillance settings. Five (38%) were positive and eight (62%) were negative. The mean turnaround time from collection to finalized result was approximately 36 h. In several negative cases, cystoscopy was deferred or avoided without any evidence of missed malignancy on follow-up, while all positive results prompted confirmatory histopathology. Clinical details and outcomes for these cases are summarized in Table 1.

### 3.2. Individual Case Summaries

*Case 1: Negative URO17^®^*

**Patient Presentation:** An 88-year-old immunocompromised male with multiple comorbidities was referred for evaluation of bladder wall thickening observed on CT imaging. He was considered high-risk for invasive procedures. **Diagnostic Workup:** Voided urine cytology was reported as suspicious of high-grade urothelial carcinoma. URO17^®^ testing returned a negative result. **Intervention and Outcome:** Considering the patient’s frailty and negative URO17^®^ result, further invasive evaluation was deferred. However, a second-opinion urologist performed cystoscopy, which revealed no abnormalities. The patient developed a multidrug-resistant urinary tract infection post-procedure, requiring hospitalization. The initial decision to forgo cystoscopy was retrospectively validated. Cystoscopy was performed approximately 10 days after the URO17^®^ result following the second-opinion referral.

*Case 2: Negative URO17^®^*

**Patient Presentation:** A 76-year-old female presented with gross hematuria. **Diagnostic Workup:** In November 2019, both voided URO17^®^ and urine cytology were negative. Repeat testing in April 2020, again yielded negative results for both assays. Renal and bladder ultrasound in April 2020, showed no abnormalities. **Intervention and Outcome:** Cystoscopy performed in May 2020, revealed a normal bladder. Vaginal atrophy and prolapse were noted as possible causes of hematuria. Catheterized URO17^®^ and cytology on the same day were also negative. The patient was managed conservatively and did not require further invasive testing. Cystoscopy took place about four weeks after the repeat URO17^®^ testing in April 2020.

*Case 3: Negative URO17^®^*

**Patient Presentation:** A 66-year-old female with a history of renal cancer and partial nephrectomy in 2017 was diagnosed with high-grade non-invasive papillary urothelial carcinoma following transurethral resection of bladder tumor (TURBT) in April 2019. The diagnosis was prompted by ultrasound findings of bladder wall thickening. The patient entered routine post-treatment surveillance. **Diagnostic Workup:** Between 2019 and 2022, the patient underwent five URO17^®^ immunocytochemical assays, performed in August 2019, December 2019 (bladder wash), September 2021, December 2021, and June 2022. All URO17^®^ results were negative. These findings were concordant with negative urine cytology and fluorescence in situ hybridization (FISH) results, including normal FISH in February 2021 and December 2021. Serial cystoscopic evaluations throughout this period, including targeted assessment of the left periureteral region, did not reveal any recurrent lesions. **Intervention and Outcome:** Although a renal and bladder ultrasound in November 2023 reported bladder wall thickening, subsequent cystoscopies through September 2024 demonstrated no evidence of recurrence. In the context of prior high-grade disease, the consistent pattern of negative URO17^®^ findings, corroborated by cytology, FISH, and cystoscopy, supported continued surveillance without the need for additional invasive intervention. URO17^®^ functioned as a rule-out diagnostic adjunct, contributing to clinical decision-making in the setting of equivocal imaging and prior malignancy. Serial cystoscopies were performed at 6- to 12-month intervals following each negative URO17^®^ test between 2019 and 2024.

*Case 4: Positive URO17^®^*

**Patient Presentation:** A 93-year-old male with a history of bladder cancer was referred for evaluation of bladder wall thickening incidentally noted on CT imaging. He had experienced multiple urinary tract infections following previous cystoscopies during surveillance. **Diagnostic Workup:** Voided urine cytology had atypical urothelial cells present. URO17^®^ immunocytochemical testing yielded a positive result. **Intervention and Outcome:** Given the patient’s advanced age and frailty, cystoscopy with biopsy was performed under anesthesia. Pathological analysis revealed high-grade T1 papillary urothelial carcinoma with concurrent carcinoma in situ (CIS). The patient was admitted postoperatively for observation. Cystoscopy with biopsy was scheduled within two weeks of the positive URO17^®^ result.

*Case 5: Positive URO17^®^*

**Patient Presentation:** A 49-year-old female with a history of intermittent microhematuria had been treated empirically with antibiotics by her gynecologist and primary care physician for presumed urinary tract infections. **Diagnostic Workup:** Voided urine cytology was negative. URO17^®^ immunocytochemical testing returned a positive result, prompting urologic referral. **Intervention and Outcome:** Cystoscopic examination revealed multifocal low-grade Ta tumors distributed along the bladder dome. The patient underwent transurethral resection of bladder tumors (TURBT), followed by perioperative administration of intravesical mitomycin. She recovered uneventfully and entered surveillance. TURBT was completed within three weeks of the positive URO17^®^ finding.

*Case 6: Positive URO17^®^*

**Patient Presentation:** A 60-year-old male presented with persistent microhematuria. Initial evaluations, including urine cytology and white-light cystoscopy, were negative for malignancy. **Diagnostic Workup:** URO17^®^ immunocytochemical testing returned a positive result. Repeat URO17^®^ testing four months later again showed a positive result, while urine cytology remained negative on both occasions. **Intervention and Outcome:** Due to persistent hematuria and repeated positive URO17^®^ findings, the patient underwent blue-light cystoscopy. Patchy fluorescence was observed on the left lateral bladder wall. Targeted biopsies confirmed carcinoma in situ (CIS). The patient was subsequently treated with standard intravesical therapy. Blue-light cystoscopy occurred roughly six weeks after the second positive URO17^®^ test.

*Case 7: Positive URO17^®^*

**Patient Presentation:** An 81-year-old male under routine surveillance for bladder cancer underwent cystoscopy in September 2019, which showed a normal bladder and bladder neck with no visible lesions. **Diagnostic Workup:** In October 2019, voided URO17^®^ testing returned a positive result. Concurrent urine cytology was negative. CT imaging in October 2019, showed asymmetric thickening of the left bladder wall, interpreted as potentially inflammatory. **Intervention and Outcome:** Due to the discordant findings, the patient underwent TURBT in December 2019. Pathology revealed high-grade non-invasive papillary urothelial carcinoma at the posterior wall and dome, with glandular differentiation. Carcinoma in situ was identified along the right lateral wall. Muscularis propria was not present in the specimen. The patient was managed according to high-risk NMIBC protocols. TURBT was undertaken approximately eight weeks after the positive URO17^®^ test, once imaging correlation was complete.

### 3.3. Interpretation of Case Findings

URO17^®^ was used in diagnostic and surveillance settings when cystoscopy, cytology, or imaging produced indeterminate or discordant findings. Results were reviewed within multidisciplinary discussions to complement existing information rather than to determine management independently. Negative findings generally supported continued observation, particularly in patients with significant comorbidities or benign imaging, while positive results prompted further evaluation, including cystoscopy or biopsy, when appropriate.

Across cases, URO17^®^ influenced the timing rather than the nature of clinical actions. During the COVID-19 pandemic, for example, limited procedural access led clinicians to use noninvasive data more strategically. In Cases 2 and 3, negative URO17^®^ findings supported short-term deferral of cystoscopy without adverse outcomes, whereas in Case 7, a positive result expedited investigation and confirmed recurrent disease. These examples highlight how test results were interpreted within the broader clinical and logistical context.

In accordance with current guideline recommendations from the American Urological Association (AUA) and the Society of Urodynamics, Female Pelvic Medicine, and Urogenital Reconstruction (SUFU) [6], follow-up timing after URO17^®^ testing was individualized rather than standardized. A positive result typically prompted additional diagnostic evaluation, such as cystoscopy or imaging, based on patient risk factors and clinician judgment. Conversely, negative findings often supported extended surveillance in low-risk or comorbid patients. This reflects real-world, risk-based decision-making in which follow-up cadence is guided by clinical context and physician discretion rather than by a fixed algorithm.

Overall, this series illustrates how URO17^®^ functioned as an adjunctive assay within established diagnostic pathways to assist interpretation when standard evaluations were inconclusive. These observations reflect pragmatic clinical judgment and workflow adaptation, offering descriptive insight into real-world test utilization rather than comparative diagnostic performance.

### 3.4. Broader Clinical Context

Bladder cancer diagnosis and surveillance remain fraught with clinical uncertainty due to limitations in traditional tools such as cystoscopy and urine cytology. These tests, while central to current practice, can yield indeterminate results that occasionally lead to delayed diagnosis or unnecessary invasive procedures. In this setting, urinary biomarkers have been explored as adjunctive tools that may help clarify inconclusive findings in selected patients.

Current AUA/SUFU clinical guidelines acknowledge the potential role of urinary biomarkers as adjuncts, particularly for patients with persistent microscopic hematuria or indeterminate cytology [6]. The present cases illustrate this principle, as URO17^®^ was incorporated into such clinical scenarios and interpreted alongside cystoscopy, cytology, and imaging results. Negative findings often supported continued observation, whereas positive results prompted further diagnostic assessment. In all instances, the assay functioned within established pathways rather than as a replacement for standard evaluations.

These observations are broadly consistent with prior studies that have evaluated URO17^®^ in larger, independent patient cohorts [5,7,8,9,10,11]. Those investigations have reported its diagnostic characteristics when applied in conjunction with traditional methods. The cited literature is provided to place these real-world observations within the broader context of urinary biomarker research and should not be interpreted as validation or extrapolation of the present findings.

### 3.5. Limitations and Future Research

This report is limited by its small size and retrospective, non-comparative design. Cases were selected for their illustrative value rather than enrolled consecutively, introducing potential selection and spectrum bias. Clinicians were aware of URO17^®^ results during management, which may have contributed to incorporation bias. These factors limit generalizability, underscoring the need for multicenter, protocol-driven studies with standardized clinical pathways to evaluate diagnostic yield, cost-effectiveness, and patient-centered outcomes such as procedure reduction and time to diagnosis. Incorporating blinded review and independent pathology validation would further strengthen reproducibility and minimize observer bias.

Preliminary evidence indicates that Keratin 17 expression may hold diagnostic value beyond bladder cancer, extending to upper tract urothelial carcinoma (UTUC). A recent tissue-based analysis of 139 UTUC cases reported 85% sensitivity and 82% specificity, with an area under the curve of 0.83 in differentiating malignant from benign urothelium [12]. Further exploration of its role in variant histologies could broaden understanding of URO17^®^’s clinical applications. In parallel, future work could examine how urinary biomarkers such as URO17^®^ might integrate into broader predictive frameworks for bladder-cancer management. Recent studies have emphasized the prognostic utility of systemic inflammatory indices and hybrid histopathologic grading models in non-muscle-invasive disease [13,14]. Although distinct from the present diagnostic focus, these developments underscore the value of combining molecular, inflammatory, and clinicopathologic data to personalize surveillance strategies.

## 4. Conclusions

The seven illustrative cases presented demonstrate how URO17^®^ immunocytochemistry can complement cystoscopy and cytology in real-world diagnostic and surveillance workflows for urothelial carcinoma. The assay’s binary, rapid results provided actionable context in cases of equivocal or negative cytology, guiding escalation or deferral of invasive procedures. These findings are descriptive and not confirmatory; prospective, protocol-driven studies are warranted to validate diagnostic accuracy, cost-effectiveness, and patient-centered outcomes.

## Figures and Tables

**Table 1:** Summary of clinical findings and URO17^®^ results.

Case	Age/Sex	Clinical Context	URO17^®^ Result(s)	Cytology	Cystoscopy/Imaging Findings	Outcome
1	88/M	Hematuria; immunocompromised	Negative (1×)	Suspicious	Normal cystoscopy	No malignancy; post-procedure UTI
2	76/F	Gross hematuria	Negative (3×)	Negative	Normal cystoscopy and ultrasound	No malignancy; managed conservatively
3	66/F	Prior HG UC; under surveillance	Negative (5×)	Negative	No recurrence on serial cystoscopies	Continued surveillance
4	93/M	Bladder wall thickening on CT	Positive (1×)	Atypical	Thickened wall; no overt lesion	HG T1 + CIS confirmed on biopsy
5	49/F	Persistent microhematuria	Positive (1×)	Negative	Multifocal non-invasive papillary lesions	TURBT + MMC
6	60/M	Persistent microhematuria	Positive (2×)	Negative	Patchy fluorescence on blue-light cystoscopy	CIS confirmed; IVT started
7	81/M	NMIBC surveillance	Positive (1×)	Negative	Asymmetric left bladder wall thickening	HG Ta + CIS; managed per high-risk NMIBC protocol

Summary of patient demographics, clinical context, URO17^®^ and cytology results, cystoscopy or imaging findings, and corresponding outcomes. Abbreviations: CIS = carcinoma in situ; CT = computed tomography; HG = high grade; IVT = intravesical therapy; MMC = mitomycin C; NMIBC = non-muscle-invasive bladder cancer; TURBT = transurethral resection of bladder tumor; UTI = urinary tract infection; HG Ta = high-grade non-invasive papillary urothelial carcinoma; HG T1 = papillary urothelial carcinoma invading the lamina propria but not the muscularis propria. (×) denotes the number of URO17^®^ tests performed per patient.

## Data Availability

Data is contained within the article.
